# Supplementary material for: Identification of KFB Family in Moso Bamboo Reveals the Potential Function of PeKFB9 Involved in Stress Response and Lignin Polymerization
Source: Int J Mol Sci. 2022 Oct 19;23(20):12568. doi: 10.3390/ijms232012568 (PMC9604269; doi:10.3390/ijms232012568)
Supplement: Supplementary file 1 [file ijms-23-12568-s001.zip › Supplementary Figures.pdf]

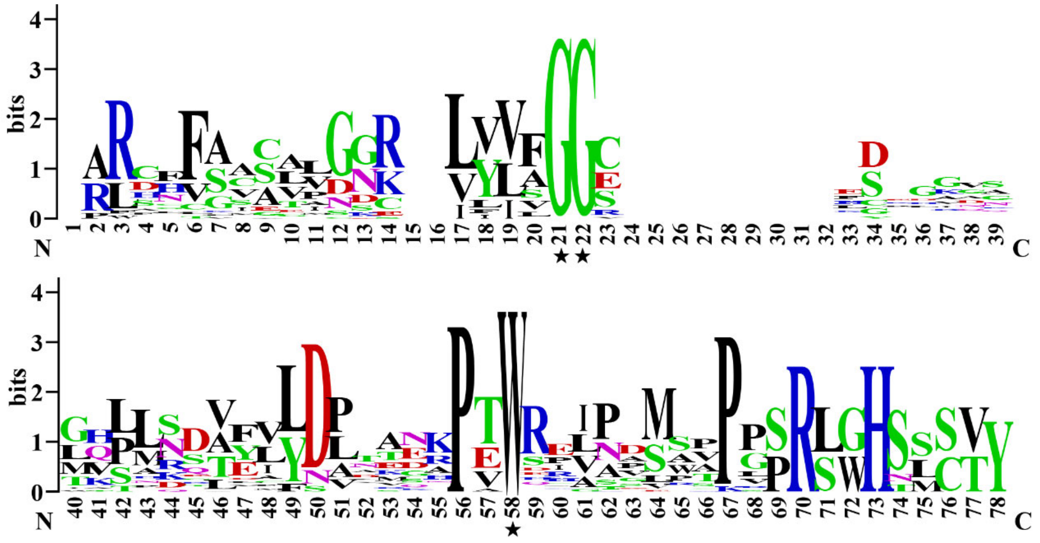

Figure S1. Consensus sequence of Kelch motif domains in PeKFBs. The overall height of each stack indicated the conservation of the sequence at that position. The conserved residues in the Kelch motif domains were marked with black asterisks.

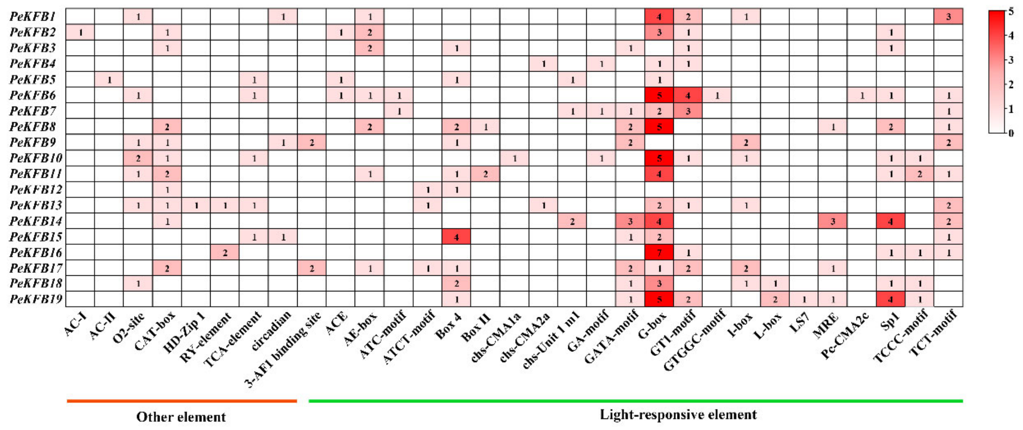

Figure S2. The analysis of conserved *cis*-elements in the promoter regions of *PeKFB*s. The right heatmap showed the number of *cis*-elements, with higher number in dark red and lower number in light red.

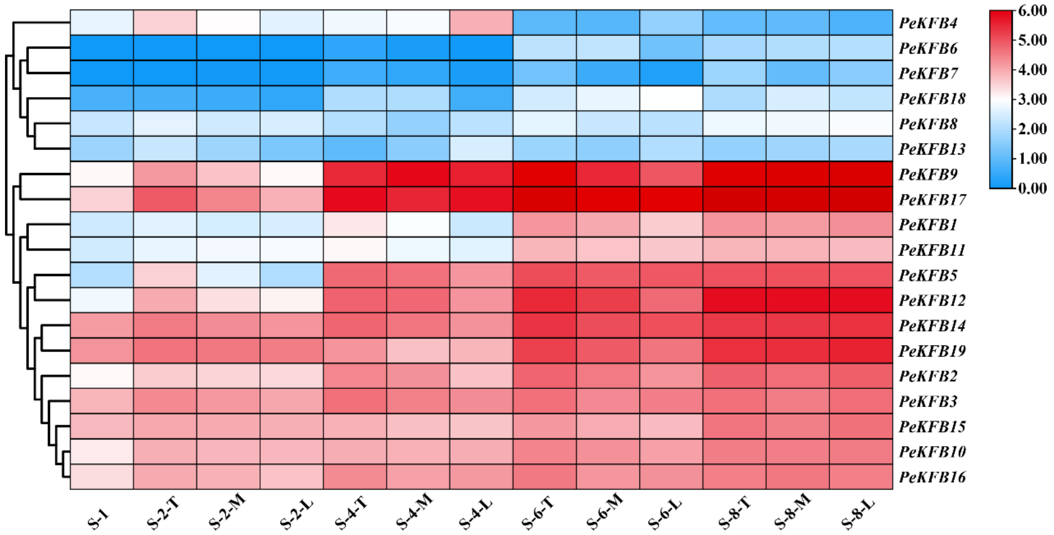

Figure S3. The expression patterns of 19 *PeKFB*s in different height shoots of moso bamboo. S-1, S-2, S-4, S-6, and S-8 represented different shoots with height of 1.0 m, 2.0 m, 4.0 m, 6.0 m, and 8.0 m, respectively. T, M, and L represented the top, middle, and lower portions of the 13th internode, respectively. The right heatmap showed gene expression, with high levels in red and low levels in blue.

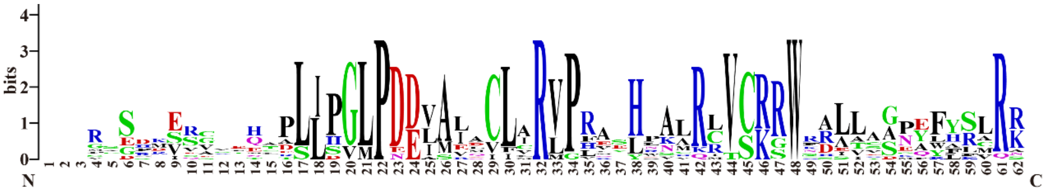

Figure S4. Consensus sequence in F-box motif domain of PeKFBs. The overall height of each stack indicated the conservation of the sequence at that position.

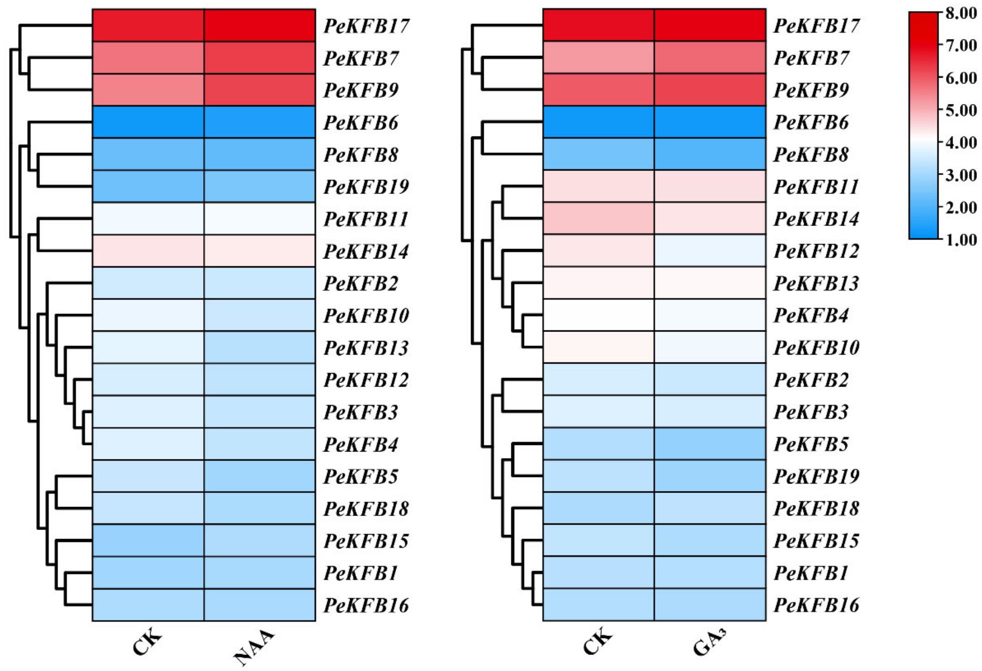

Figure S5. Expression analysis of *PeKFB*s in leaves of moso bamboo under NAA and GA_3 treatments. The right heatmap showed gene expression, with high levels in red and low levels in blue.
